# Corticosteroid treatment for early acute respiratory distress syndrome: a systematic review and meta-analysis of randomized trials

**DOI:** 10.1186/s40560-020-00510-y

**Published:** 2020-12-07

**Authors:** Yohei Hirano, Shunsuke Madokoro, Yutaka Kondo, Ken Okamoto, Hiroshi Tanaka

**Affiliations:** Department of Emergency and Critical Care Medicine, Juntendo University Urayasu Hospital, 2-1-1 Tomioka, Urayasu, Chiba 279-0021 Japan

**Keywords:** Corticosteroids, Acute respiratory distress syndrome, Systematic reviews, Meta-analysis

## Abstract

**Background:**

The effect of corticosteroid treatment on survival outcome in early acute respiratory distress syndrome (ARDS) is still debated. We conducted a systematic review and meta-analysis of randomized controlled trials (RCTs) to assess the efficacy of prolonged corticosteroid therapy in early ARDS.

**Methods:**

We assessed the MEDLINE, Cochrane Central Register of Controlled Trials, and Web of Science databases from inception to August 1, 2020. We included RCTs that compared prolonged corticosteroid therapy with control treatment wherein the intervention was started within 72 h of ARDS diagnosis. Two investigators independently screened the citations and conducted the data extraction. The primary outcomes were all-cause 28- or 30-day mortality and 60-day mortality. Several endpoints such as ventilator-free days and adverse events were set as the secondary outcomes. DerSimonian-Laird random-effects models were used to report pooled odds ratios (ORs).

**Results:**

Among the 4 RCTs included, all referred to the all-cause 28- or 30-day mortality. In the corticosteroid group, 108 of 385 patients (28.1%) died, while 139 of 357 (38.9%) died in the control group (pooled OR, 0.61; 95% confidence interval [CI], 0.44–0.85). Three RCTs mentioned the all-cause 60-day mortality. In the corticosteroid group, 78 of 300 patients (26.0%) died, while 101 of 265 (38.1%) died in the control group (pooled OR, 0.57; 95% CI, 0.40–0.83). For secondary outcomes, corticosteroid treatment versus control significantly prolonged the ventilator-free days (4 RCTs: mean difference, 3.74; 95% CI, 1.53–5.95) but caused hyperglycemia (3 RCTs: pooled OR, 1.52; 95% CI, 1.04–2.21).

**Conclusions:**

Prolonged corticosteroid treatment in early ARDS improved the survival outcomes.

**Trial registration:**

PROSPERO, CRD42020195969

**Supplementary Information:**

The online version contains supplementary material available at 10.1186/s40560-020-00510-y.

## Background

Acute respiratory distress syndrome (ARDS) is characterized by an acute diffuse, inflammatory lung injury with an increased alveolar-capillary permeability and loss of aerated lung tissue, contributing to impaired oxygenation [1]. ARDS is a challenging disease for practitioners in intensive care due to the lack of effective therapeutics and its high mortality rate.

As ARDS is an inflammatory syndrome, anti-inflammatory drugs including corticosteroids might serve as potential therapeutics for ARDS. Until now, there have been a number of randomized controlled studies conducted to assess the efficacy of corticosteroids on ARDS [2–7]. However, its effectiveness on patient survival is still controversial.

Data analysis of individual patients from 4 randomized controlled trials (RCTs) investigating prolonged methylprednisolone therapy in early and late ARDS revealed a reduction in hospital mortality [8]. After 9 RCTs determined the effectiveness of corticosteroid treatment, the latest guidelines by the Society of Critical Care Medicine and the European Society of Intensive Care Medicine made a conditional recommendation of the use of corticosteroids in patients with moderate to severe ARDS within 14 days from onset [9]. However, this recommendation is partly based on RCTs, which included severe community-acquired pneumonia instead of ARDS, and this could be an ascertainment bias [10]. The recent systematic reviews and meta-analysis of pharmacological agents for adults with ARDS could not show the survival benefit of corticosteroid use [11].

It is possible that prolonged corticosteroid treatment may have a better therapeutic effect when used in the very early phase of ARDS due to its ability to reduce hyperinflammation and fibrosis. Therefore, we aimed to conduct a systematic review and meta-analysis of the present RCTs to assess the efficacy of prolonged corticosteroid therapy started in the early phase of ARDS.

## Methods

### Data sources and search strategies

To identify eligible trials, we searched the Cochrane Central Register of Controlled Trials, MEDLINE, and the Web of Science databases on June 23, 2020. The search was repeated to detect recent publications until August 1, 2020. Searches were not restricted by publication status, date, and sample size. The details of search terms and strategy employed are described in electronic supplementary material 1 (Additional file 1). The present study was registered in the PROSPERO database (CRD42020195969).

### Study selection

Titles and abstracts of the trials were retrieved from the databases. After all duplicate studies were excluded, two investigators (YH and SM) independently screened the titles and abstracts for eligibility. When a disagreement was identified between reviewers, the full text of the article was obtained to determine the study’s eligibility, and differences in opinion were resolved by consensus. If disagreements could not be reconciled, a third investigator (YK) was consulted. The full texts of articles included in the final selection were independently reviewed by two investigators (YH and SM). Finally, eligible studies were determined after discussion and resolution of discrepancies by consensus.

We identified the eligible studies by following a research question formulated according to the participants, interventions, comparisons, and outcomes model, which is “P” for adult (≥ 18 years old) patients with a diagnosis of ARDS, “I” for prolonged corticosteroids administration started within 72 h from diagnosis of ARDS, “C” for no prolonged corticosteroids administration (control or placebo), and “O” for all-cause mortality. “Prolonged” corticosteroid intervention was defined as corticosteroid administration for two consecutive days or more. We did not restrict the definition of ARDS to the latest definition [12]; instead, we considered all past definitions of ARDS when identifying the eligible studies.

### Data extraction

Data were extracted independently by two investigators, and consensus was reached. The data extracted included the following: authors, publication year, country, study design, number of sites, inclusion period, the completion of the trials, number and participant details including age and gender, severity of patients such as sequential organ failure assessment score, lung injury score, oxygenation level, ventilator setting and strategy, cause of ARDS, exclusion criteria of the study, timing of initial intervention, type of corticosteroids, intervention protocol, and the study results.

### Study endpoints

Twenty-eight- or 30-day mortality and 60-day mortality were set as the primary outcomes. The secondary outcomes were ventilator-free days at 28 days, partial pressure of oxygen in arterial blood (PaO_2_)/fraction of inspired oxygen (FiO_2_) on days 6 or 7, and adverse events including hyperglycemia, nosocomial infections, and gastrointestinal bleeding.

### Subgroup analysis

Moderate to severe ARDS were independently assessed as a subgroup using subgroup analysis. For this analysis, only primary outcomes were compared.

### Assessment of methodological quality

We adapted the Cochrane risk-of-bias tool to assess the quality of the studies included for meta-analysis [13]. Two investigators (YH and SM) independently assessed the risk of bias of the included studies, and a third investigator (YK) resolved the discrepancies using an independent blinded evaluation. Additionally, we graded the quality of evidence of each finding based on the criteria established by the Grading of Recommendations Assessment, Development and Evaluation working group [14]. The quality of the study methodology was independently classified by the two investigators as high, intermediate, low, or very low, based on study design, risk of bias, indirectness, inconsistency, imprecision, and publication bias. The publication biases were assessed visually by inspecting the funnel plots as well as analytical appraisals based on Egger’s linear regression test [15]. A two-sided *p* value of 0.10 was considered significant in Egger’s linear regression test.

### Statistical analysis

We pooled the eligible patients for each outcome using the DerSimonian-Laird random-effects model with weights. For dichotomous outcomes, we calculated the odds ratios (ORs) with 95% confidence intervals (CIs) using the Mantel-Haenszel method. For continuous outcomes, the mean differences (MDs) with 95% CIs were calculated using the inverse variance method. We verified the heterogeneity of the studies using the Cochran chi-squared, tau-squared, and *I*^2^ statistics (*I*^2^ > 50% was considered as a measure of severe heterogeneity). We applied unadjusted *p* values for the significance assessment in this study, which were set at the two-tailed 0.05 level for hypothesis testing and at the 0.10 level for heterogeneity testing. All statistical analyses were performed using the Cochrane systematic review software Review Manager version 5.3.5 for Mac (the Nordic Cochrane Centre, the Cochrane Collaboration, Copenhagen, Denmark), except for the analysis of publication bias, which was analyzed by ProMeta 3.0 (https://idostatistics.com/prometa3/).

## Results

### Search results

**Fig. 1:**
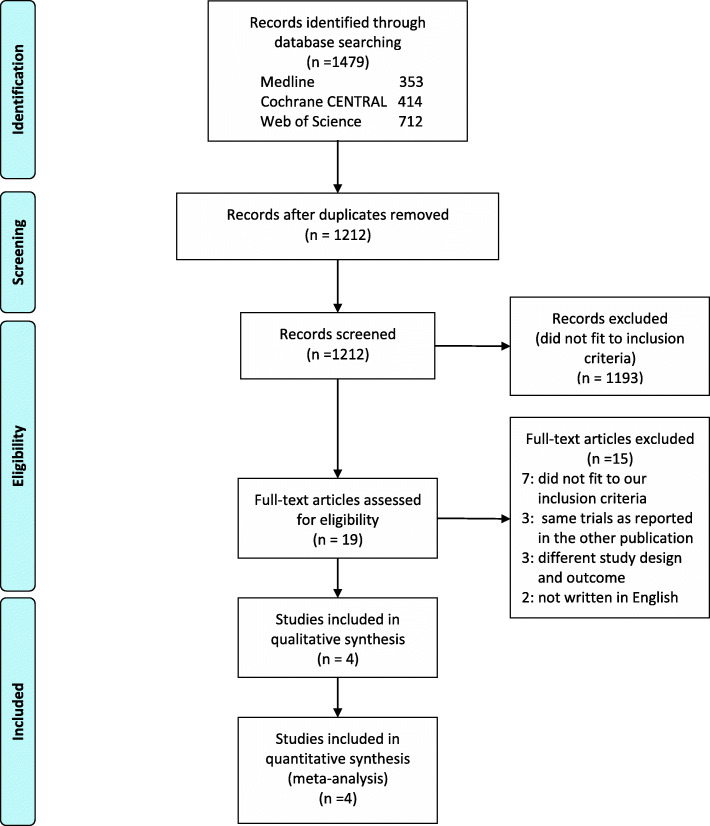
Flow diagram of the search strategy and study selection

After the elimination of duplicates, we identified 1212 studies from the electronic databases. Among them, only 19 studies were eligible based on the assessment of the study title and abstract. After the review of their full-text articles, 15 studies were excluded because they did not meet the inclusion criteria (i.e., participants with severe pneumonia or late ARDS), they were the same trials as reported in the other publications, they were conducted with a different study design or outcome, or they were not fully available in English. Finally, 4 RCTs were included in this meta-analysis [3, 6, 7, 16] (Fig. 1).

### Study characteristics

We analyzed a total of 742 patients from 4 RCTs. Among them, 385 patients were randomly assigned to the corticosteroids group and 357 to the control group. The individual characteristics of the 4 RCTs are detailed in Additional file 2. Three of the four studies were multicentric studies. Two RCTs included moderate or severe ARDS patients, while sepsis-induced ARDS participants were included in the other two trials. Pneumonia was the primary cause of ARDS, which occurred in 52.7% of the participants (391 out of 742). Patients who had received corticosteroids before randomization were excluded in all the trials. Three in four trials complied with the lung-protective strategy (low tidal volume with positive end-expiratory pressure). There was a variation of the type or the schedule of corticosteroid treatment among studies.

### Outcome

**Fig. 2:**
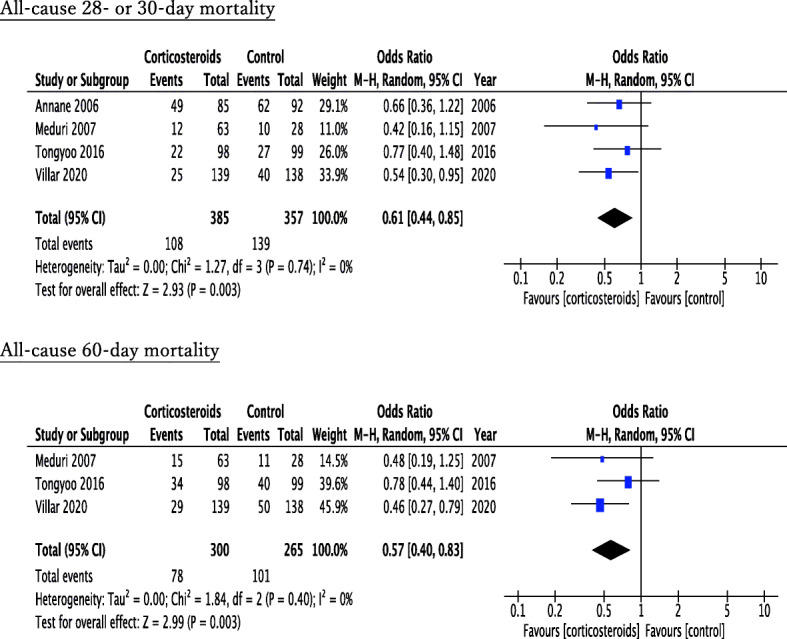
Forest plot of the 28- or 30-day and 60-day mortality in the comparison between corticosteroid treatment and control in early ARDS

**Fig. 3:**
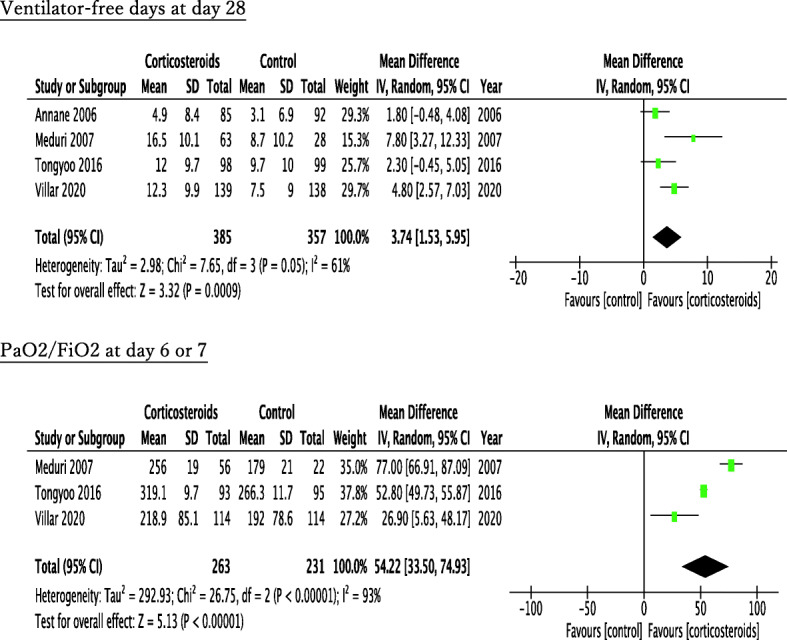
Forest plot of the ventilator-free days at 28 days and partial pressure of oxygen in arterial blood (PaO_2_)/fraction of inspired oxygen (FiO_2_) at days 6 or 7 in the comparison between corticosteroid treatment and control in early ARDS

**Fig. 4:**
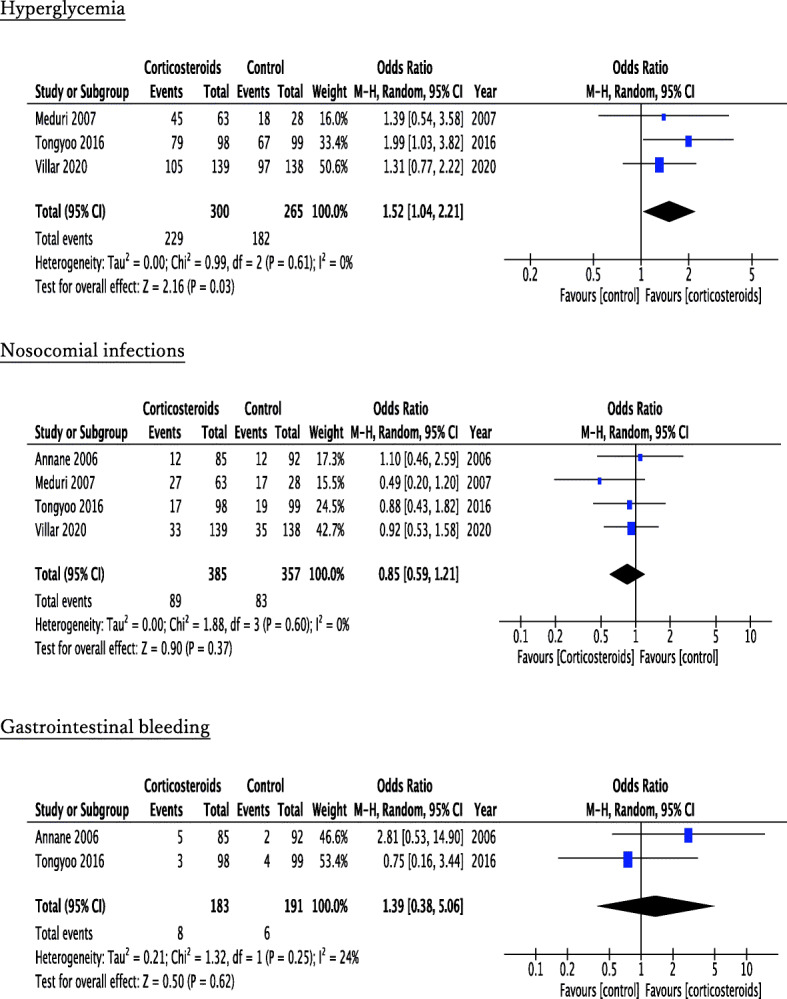
Forest plot of the adverse events (hyperglycemia, nosocomial infections, and gastrointestinal bleeding) in the comparison between corticosteroid treatment and control in early ARDS

The forest plot of the primary outcomes is shown in Fig. 2. Four RCTs referred to all-cause mortality at 28 or 30 days and three RCTs at 60 days. Within 28–30 hospital days, 108 of 385 patients (28.1%) died in the corticosteroid treatment group, while 139 of 357 patients (38.9%) died in the control group. Corticosteroid treatment showed significant improvement in 28- or 30-day mortality (pooled OR, 0.61; 95% CI, 0.44–0.85). Within 60 hospital days, 78 of 300 patients (26.0%) died in the corticosteroid group, while 101 of 265 patients (38.1%) died in the control group. Similar to that in 28- or 30-day mortality, corticosteroid therapy also demonstrated significant improvement in 60-day mortality (pooled OR, 0.57; 95% CI, 0.40–0.83). Evaluation of the secondary outcomes manifested prolongation of ventilator-free days at day 28 and improvement of PaO_2/_FiO_2_ at day 6 or day 7 in the corticosteroid group compared with the control group (ventilator-free days: pooled MD, 3.74; 95% CI, 1.53–5.95; PaO_2/_FiO_2_: pooled MD, 54.22; 95% CI, 33.50–74.93) (Fig. 3). Among the adverse events, corticosteroids resulted in significant hyperglycemia (pooled OR, 1.52; 95% CI, 1.04–2.21), whereas there was no significant difference in nosocomial infections or gastrointestinal bleeding (nosocomial infections: pooled OR, 0.85; 95% CI, 0.59–1.21; gastrointestinal bleeding: pooled OR, 1.39; 95% CI, 0.38–5.06) (Fig. 4).

We also analyzed the subgroup of moderate to severe ARDS patients. Three in four RCTs were included in the analysis. Corticosteroid treatment presented a significant improvement in 28- or 30-day as well as 60-day mortality compared with control even in the moderate to severe ARDS population (28- or 30-day mortality: pooled OR, 0.58; 95% CI, 0.38–0.87; 60-day mortality: pooled OR, 0.54; 95% CI, 0.36–0.79) (Additional file 3).

### Heterogeneity

For the primary outcomes, heterogeneity was not observed among the studies (28- or 30-day mortality: *I*^2^ = 0%, tau^2^ = 0.00, *χ*^2^ = 1.27, *p* = 0.74; 60-day mortality: *I*^2^ = 0%, tau^2^ = 0.00, *χ*^2^ = 1.84, *p* = 0.40) (Fig. 2). The evaluation of heterogeneity for other outcomes is described in forest plots (Figs. 3 and 4, Additional file 3).

### Publication bias, risk of bias, and quality of evidence

We also analyzed the presence of publication bias for the primary outcomes. A visual inspection of the funnel plot and Egger’s linear regression test failed to show the existence of publication bias in both 28- or 30-day and 60-day mortality (*p* = 0.529 and *p* = 0.904, respectively) (Additional file 4). Regarding the risk of bias, the blinding of participants and personnel was categorized as high risk in one RCT from Villar et al. because clinicians were aware of the group assignment in the trial. We also evaluated that there was another high risk of bias in this RCT due to the early termination of the study (Additional files 5 and 6).

The summary of findings is detailed in Table 1. For the effect of corticosteroids on the primary outcomes, the quality of evidence was rated as moderate. The grade was lowered by 1 point because different types of corticosteroids and treatment regimens among studies were regarded as indirect evidence.

**Table 1:** Summary of findings

Outcomes	Anticipated absolute effects (95% CI)		Relative effect (OR (95% CI))	No. of participants (no. of studies)	Certainty of evidence (GRADE)
	Control	Risk difference with corticosteroids			
All-cause 28- or 30-day mortality	389 per 1000	109 fewer per 1000 (170 fewer to 38 fewer)	0.61 (044–0.85)	742 (4 RCTs)	Moderate^a^
All-cause 60-day mortality	381 per 1000	121 fewer per 1000 (183 fewer to 43 fewer)	0.57 (040–0.83)	565 (3 RCTs)	Moderate^a^
Ventilator-free days at 28 days		MD 3.74 more (1.53 more to 5.95 more)		742 (4 RCTs)	Low^a,b^
PaO_2_/FiO_2_ at days 6 or 7		MD 54.22 more (33.5 more to 74.93 more)		494 (3 RCTs)	Very low^a,c^
Hyperglycemia	687 per 1000	82 more per 1000 (8 more to 142 more)	1.52 (1.04–2.21)	565 (3 RCTs)	Moderate^a^
Nosocomial infections	232 per 1000	28 fewer per 1000 (81 fewer to 36 more)	0.85 (0.59–1.21)	742 (4 RCTs)	Low^a,d^
Gastrointestinal bleeding	31 per 1000	12 more per 1000 (19 fewer to 110 more)	1.39 (0.38–5.06)	374 (2 RCTs)	Low^a,d^

Grades of evidence according to the Grading of Recommendations Assessment, Development and Evaluation (GRADE) working group:

High certainty—We are very confident that the true effect lies close to that of the estimate of the effect

Moderate certainty—We are moderately confident in the effect estimate. The true effect is likely to be close to the estimate of the effect, but there is a possibility that it is substantially different

Low certainty—Our confidence in the effect estimate is limited. The true effect may be substantially different from the estimate of the effect

Very low certainty—We have very little confidence in the effect estimate. The true effect is likely to be substantially different from the estimate of the effect

*PaO*_*2*_ partial pressure of oxygen in arterial blood, *FiO*_*2*_ fraction of inspired oxygen

^a^Different types of corticosteroids and treatment regimens among studies were regarded as indirectness of evidence. Downgraded by 1

^b^Heterogeneity observed among the studies (*I*^2^ = 61.0%, *χ*^2^ = 7.65, *p* = 0.05) was regarded as inconsistency of evidence. Downgraded by 1

^c^Heterogeneity observed among the studies (*I*^2^ = 93.0%, *χ*^2^ = 26.75, *p* < 0.001) was regarded as inconsistency of evidence. Downgraded by 1

^d^Imprecision was observed among the studies. Downgraded by 1

## Discussion

The current study is the most recent systematic review and meta-analysis of RCTs investigating the efficacy of early phase corticosteroid treatment on ARDS. The results suggest that prolonged corticosteroid treatment initiated within 72 h of ARDS diagnosis showed improvement in mortality (short term of 28- or 30-day as well as 60-day), in comparison with control.

Corticosteroids are potent therapeutic agents for a variety of overactive inflammatory diseases due to their anti-inflammatory properties including maintaining endothelial integrity and reduction of pro-inflammatory cytokines and nitric oxide synthase. They also inhibit fibroblast growth and collagen deposition, thus having antifibrotic properties [17]. Histologically, ARDS is considered to be diffuse alveolar damage caused by a sequential process of hyperinflammation, the so-called exudative, proliferative, and fibrotic phase [18, 19]. Therefore, corticosteroids may have therapeutic potential for the management of ARDS [17]. However, the use of corticosteroids in clinical medicine has occasionally witnessed adverse effects such as vulnerability to infection, hyperglycemia, and gastrointestinal ulceration or bleeding, which may worsen the outcome of patients [20]. Until now, there have been several clinical trials conducted to investigate the efficacy of corticosteroids on ARDS.

A single high dose of corticosteroids was first tested on ARDS by an RCT in 1987, but they did not manifest beneficial effects on survival [21]. Based on this result, recent studies have examined a lower dose of prolonged corticosteroid therapy on ARDS. Two RCTs examined the effect of a lower dose of corticosteroids on persistent ARDS, although the results were in the opposite direction [4, 5]. A small RCT by Meduri et al. suggested improvement in hospital mortality, while another RCT by Steinberg et al. demonstrated no beneficial effect of corticosteroids on mortality in late ARDS. They also showed that corticosteroids were associated with a significant increase in 60- and 180-day mortality rates in patients enrolled after at least 14 days from the onset of ARDS. Thus, the recent RCTs have focused on corticosteroid therapy in early ARDS, which is the target population in the current meta-analysis.

Several systematic reviews and meta-analysis have been conducted on corticosteroid treatment in ARDS. However, their benefits on survival outcome are conflicting. This controversy might be caused by diversity in the inclusion of ARDS patients. Based on the analysis of 9 RCTs that proved the survival benefit of corticosteroid treatment, the guidelines by the Society of Critical Care Medicine and European Society of Intensive Care Medicine for the diagnosis and management of critical illness-related corticosteroid insufficiency in gravely ill patients suggest the use of corticosteroids in moderate to severe ARDS patients within 14 days from disease onset [9, 10]. However, caution is required in interpreting the result because 2 out of the 9 trials targeted patients with community-acquired pneumonia (CAP), even if pneumonia is the prime cause of ARDS [22, 23]. The recent Cochrane review for pharmacological agents for adults with ARDS did not include CAP patients, and they did not witness any significant improvement in mortality after corticosteroid therapy [11]. In addition, considering the sequential process of inflammation in ARDS, i.e., the exudative, proliferative, and fibrotic phase [18, 19], the timing of initiation of corticosteroid therapy may be important to improve outcomes. The previous meta-analyses have included both early and late phases of ARDS [8, 9, 11]. There is substantial evidence in literature stating that the early initiation of methylprednisolone treatment was associated with faster disease resolution compared with late initiation (> 7 days) [24].

The current meta-analysis demonstrates its importance because we included only the ARDS population in which corticosteroid treatment was initiated within 72 h of disease diagnosis. A recent RCT comparing dexamethasone with placebo in early ARDS has also been included, which was not analyzed in the previous meta-analysis [7]. We did not include RCTs that examined CAP or COVID-19 patients. However, it should be noted that a very recent nationwide RCT showed the benefit of dexamethasone treatment in severe COVID-19 patients receiving invasive mechanical ventilation that significantly reduced death by one third [25]. While our results revealed that the early induction of corticosteroids causes hyperglycemia, we manifested that the favorable improvement of short-term survival was the primary outcome. The survival benefit of corticosteroid treatment was also retained in the subgroup of early and moderate to severe ARDS. However, we could not specify the efficacy of corticosteroids in mild ARDS because we were unable to perform subgroup analysis for this population due to the lack of RCTs on them.

### Limitations of the study

This meta-analysis has several limitations. First, there might be publication bias. Although our visual inspection of the funnel plot and Egger’s linear regression test failed to show the existence of publication bias in primary outcomes, their reliability is very weak because our analysis included only 4 RCTs for the primary outcomes. Second, included RCTs have some methodological concerns. In the largest RCT by Villar et al., only 27% of eligible patients were enrolled in the study and clinicians and investigators completing the outcome assessment were aware of the group allocation. These problems might cause selection and performance bias. Moreover, there was an early termination of the study due to the low enrollment rate in this study, which reduced the confidence of the results. Third, there was diversity in the inclusion criteria of ARDS patients such as severity or cause of ARDS. It should be noted that corticosteroids are conditionally recommended for septic shock patients that is not responsive to fluid and vasopressor therapy [9]. As participants of the included two trials have ARDS with severe sepsis or septic shock, the result of our analysis might be partially affected by the beneficial effect of corticosteroids on septic shock. In addition, there were variations in the ARDS treatment strategy because lung-protective ventilation for ARDS was partially or never performed in two trials [26]. Treatment protocol, amounts, and types of corticosteroids used were also different among studies. Heterogeneity is the weakness of meta-analysis. However, our analysis focused on a limited population of ARDS compared with a previous meta-analysis with considerable heterogeneity. We also added the subgroup analysis of moderate to severe ARDS patients, which did not change the results of beneficial survival outcomes by corticosteroids. Last, our meta-analysis did not clarify the effect of corticosteroids on long-term survival outcomes. Among the adverse events assessed, only a significant increase in hyperglycemia was found. However, corticosteroid use causes other types of adverse events such as myopathy or intensive care unit-acquired weakness [20]. Therefore, long-term survival with high quality of life is our most important outcome.

## Conclusions

Based on the findings of this meta-analysis, prolonged corticosteroid treatment initiated within 72 h of ARDS diagnosis improves short-term survival. We therefore recommend the use of corticosteroids in the early phase of ARDS. However, there are concerns that need to be addressed such as the beneficial effect on long-term survival outcomes, difference of the effect among the diverse cause of ARDS, or the type, amount, and duration of corticosteroids to be used. These questions should be elucidated in future trials.

## Supplementary Information

**Additional file 1.** The search terms and strategy of the current study.

**Additional file 2.** Details of the included studies.

**Additional file 3.** Forest plot of the 28- or 30-day and 60-day mortality in comparison between corticosteroids treatment and control in the subgroup of early moderate to severe ARDS.

**Additional file 4.** Funnel plot of the 28- or 30-day and 60-day mortality in comparison between corticosteroid treatment and control in early ARDS.

**Additional file 5.** Risk of bias summary.

**Additional file 6.** Risk of bias graph.

## Data Availability

The data and material used for this meta-analysis are contained in the references.

## Abbreviations

ARDS: Acute respiratory distress syndrome
CAP: Community-acquired pneumonia
CI: Confidence interval
FiO_2_: Fraction of inspired oxygen
MD: Mean difference
OR: Odds ratios
PaO_2_: Partial pressure of oxygen in arterial blood
RCTs: Randomized controlled trials
