# Supplementary material for: Corticosteroid treatment for early acute respiratory distress syndrome: a systematic review and meta-analysis of randomized trials
Source: J Intensive Care. 2020 Dec 7;8:91. doi: 10.1186/s40560-020-00510-y (PMC7720037; doi:10.1186/s40560-020-00510-y)
Supplement: Supplementary file 1 — Additional file 1. The search terms and strategy of the current study. [file 40560_2020_510_MOESM1_ESM.docx]

**MEDLINE:**

| #1 | **(''respiratory distress syndrome''[mesh]) OR (''respiratory distress syndrome''　[tiab]))** |
| --- | --- |
| #2 | **(''acute lung injury'' [mesh]) OR (''acute lung injury'' [tiab])** |
| #3 | #1 OR #2 |
| #4 | **(Glucocorticoids[mesh]) OR (Glucocorticoids[tiab]) OR (hydrocortisone[mesh]) OR (hydrocortisone[tiab]) OR (prednisolone[mesh]) OR (prednisolone[tiab]) OR (methylprednisolone[tiab]) OR (dexamethasone[mesh]) OR (dexamethasone[tiab])** |
| #5 | #3 AND #4 |
| #6 | **(''randomized controlled trial'' [pt]) OR (''controlled clinical trial'' [pt]) OR (randomized[tiab]) OR (placebo[tiab]) OR (randomly[tiab]) OR (trial[tiab]) NOT (animals [mh] NOT humans [mh])** |
| #7 | #5 AND #6 |

**Cochrane Central Register of Controlled Trials:**

| #1 | MeSH descriptor: [Respiratory Distress Syndrome, Adult] explode all trees |
| --- | --- |
| #2 | (ARDS): ti, ab, kw |
| #3 | MeSH descriptor: [Acute Lung Injury] explode all trees |
| #4 | (acute lung injury): ti, ab, kw |
| #5 | #1 OR #2 OR #3 OR #4 OR #5 |
| #6 | MeSH descriptor: [Glucocorticoids] explode all trees |
| #7 | (glucocorticoid): ti, ab, kw |
| #8 | MeSH descriptor: [Hydrocortisone] explode all trees |
| #9 | (hydrocortisone): ti, ab, kw |
| #10 | MeSH descriptor: [Prednisolone] explode all trees |
| #11 | (prednisolone): ti, ab, kw |
| #12 | MeSH descriptor: [Methylprednisolone] explode all trees |
| #13 | (methylprednisolone): ti, ab, kw |
| #14 | MeSH descriptor: [Dexamethasone] explode all trees |
| #15 | (dexamethasone): ti, ab, kw |
| #16 | #7 OR #8 OR #9 OR #10 OR #11 OR #12 OR #13 OR #14 OR #15 OR #16 |
| #17 | #6 AND #17 |
| #18 | Trials |

**Web of Science:**

| #1 | **ALL=''respiratory distress syndrome''** |
| --- | --- |
| #2 | **ALL=’'acute lung injury’’** |
| #3 | #1 OR #2 |
| #4 | **ALL=''glucocorticoids''** |
| #5 | **ALL= ''hydrocortisone''** |
| #6 | **ALL=''prednisolone''** |
| #7 | **ALL=''methylprednisolone''** |
| #8 | **ALL=''dexamethasone''** |
| #9 | #4 OR #5 OR #6 OR #7 OR #8 |
| #10 | #3 AND #9 |
| #11 | ALL=**''**randomized controlled trial**''** OR ALL=**''**controlled clinical trial**''** OR ALL=**''**randomized**''** OR ALL=**''**placebo**''** OR ALL=**''**drug therapy**''** OR ALL=**''**randomly**''** OR ALL=**''**trial**''** OR ALL=**''**groups**''** |
| #12 | #10 AND #11 |
