# Supplementary material for: Corticosteroid treatment for early acute respiratory distress syndrome: a systematic review and meta-analysis of randomized trials
Source: J Intensive Care. 2020 Dec 7;8:91. doi: 10.1186/s40560-020-00510-y (PMC7720037; doi:10.1186/s40560-020-00510-y)
Supplement: Supplementary file 2 — Additional file 2. Details of the included studies. [file 40560_2020_510_MOESM2_ESM.docx]

| First author, year | Villar, 2020 | Tongyoo, 2016 | Meduri, 2007 | Annane 2006 |
| --- | --- | --- | --- | --- |
| Country | Spain | Thailand | United States | France |
| No of site | 17 | 1 | 5 | 19 |
| Inclusion period | 2013-2018 | 2010-2014 | 1997-2002 | 1995-1999 |
| The completion of the trial | No (low enrollment rate) | Yes | Yes | Yes |
| No of patients | 277 | 197 | 91 | 177 |
| Type of patients | Moderate to severe ARDS  (moderate;86.3%, severe; 13.7%) | ARDS with severe sepsis  (mild; 33.0%, moderate to severe; 67.0%) | Severe ARDS | ARDS with septic shock  (post hoc analysis of RCT) |
| Age of patients (years)*  (intervention vs control) | 56 (14) vs 58 (15) | 64.5 (17.3) vs 64.3 (16.0) | 50.1 (15.3) vs 53.2 (15.3) | 61.0 (16.0) vs 59.0 (18.0) |
| Gender (male; %) | 69.0 | 51.3 | 51.6 | 68.4 |
| SOFA score *  (intervention vs control) | 8.7 (3.1) vs 8.6 (3.1) | 10.9 (3.5) vs 10.8 (3.6) | Unknown | Unknown |
| LIS *  (intervention vs control) | Unknown | 2.2 (0.9) vs 2.2 (1.0) | 3.2 (0.4) vs 3.1 (0.4) | Unknown |
| PaO_2_/FiO_2_ (mmHg)*  (intervention vs control) | 142.4 (37.3) vs 143.5 (33.4) | 175.4 (6.9) vs 172.4 (6.7) | 118.4 (51.2) vs 125.9 (38.6) | 104.0 (42.0) vs 108 (45.0) |
| PEEP (cmH2O) *  (intervention vs control) | 11.9 (3.3) vs 12.1 (3.3) | 7.3 (3.0) vs 6.8 (2.5) | 13.0 (5.0) vs 11.2 (4.0) | 6.8 (2.7) vs 7.4 (3.0) |
| Lung-protective  mechanical ventilation | Yes | Yes | Partially Yes | No |
| Cause of ARDS (%)  Pneumonia  Sepsis  Trauma | 53.1  24.2  7.6 | 50.8  100  0 | 41.8  67.0  Unknown | 59.9  100  ０ |
| Exclusion criteria | Pregnancy or lactation  Brain death  Terminal-stage of diseases  Decision to DNR  Corticosteroids treatment  Immunosuppressive drugs  Enrollment in another trial  Severe COPD  Congestive heart failure | Pregnancy  Moribund state  Terminal-stage of diseases  Immunosuppressive drugs  Corticosteroids treatment  Severe diabetes | Moribund state  Terminal-stage of diseases  Active tuberculosis  Corticosteroids treatment  Burns  HIV positive  Gastrointestinal bleeding  Organ transplant recipients  Severe liver disease | Pregnancy  Advanced cancer  Corticosteroids treatment  HIV positive  Myocardial infarction  Pulmonary embolism |
| Timing of initial intervention* | At 24h after ARDS onset  (No later than 30h after ARDS onset) | Intervention; 11.9 (7.9) h vs.  control;14.0 (10.2) h  from meeting severe sepsis criteria | Within 72 h of ARDS diagnosis | Within 8 h of the onset of shock |
| Type of corticosteroids | Dexamethasone | Hydrocortisone | Methylprednisolone | Hydrocortisone |
| Intervention protocol | 20mg (day1 to day5)  10mg (day6 to day10) | 50mg every 6h (day1 to day7) | 1mg/kg (day1 to day14)  0.5mg/kg (day15 to day21)  0.25mg/kg (day22 to day25)  0.125mg/kg (day26 to day28) | 50mg every 6h (day1 to day7)  with 50 μg of fludrocortisone  (once a day) |

ARDS; acute respiratory distress syndrome, RCT; randomized controlled trial, SOFA; sequential organ failure assessment, LIS; lung injury score, PaO_2_; partial pressure of arterial oxygen, FiO_2_; fraction of inspiratory oxygen, PEEP; positive end-expiratory pressure, DNR; do-not-resuscitate, COPD; chronic obstructive pulmonary disease, HIV; human immunodeficiency virus. *: continuous variables are shown as mean (standard deviation), if applicable.
