# Supplementary material for: Corticosteroid treatment for early acute respiratory distress syndrome: a systematic review and meta-analysis of randomized trials
Source: J Intensive Care. 2020 Dec 7;8:91. doi: 10.1186/s40560-020-00510-y (PMC7720037; doi:10.1186/s40560-020-00510-y)
Supplement: Supplementary file 3 — Additional file 3. Forest plot of the 28- or 30-day and 60-day mortality in comparison between corticosteroids treatment and control in the subgroup of early moderate to severe ARDS. [file 40560_2020_510_MOESM3_ESM.docx]

All-cause 28- or 30-day mortality

All-cause 60-day mortality
