# Supplementary figures and images for: Corticosteroid treatment for early acute respiratory distress syndrome: a systematic review and meta-analysis of randomized trials
Source: J Intensive Care. 2020 Dec 7;8:91. doi: 10.1186/s40560-020-00510-y (PMC7720037; doi:10.1186/s40560-020-00510-y)

All-cause 28- or 30-day mortality


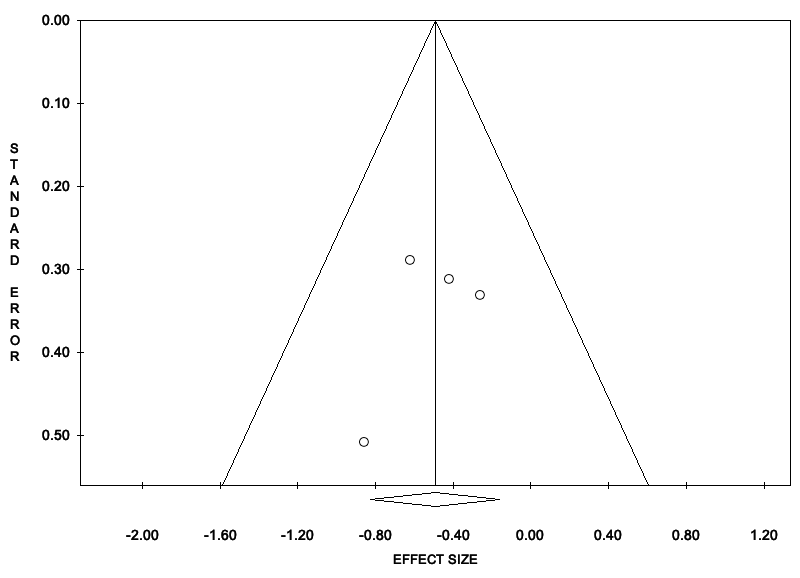


All-cause 60-day mortality


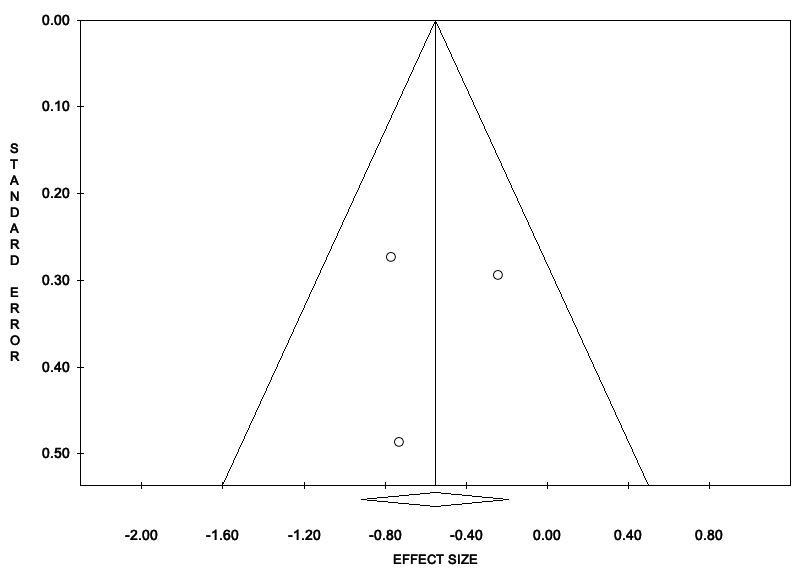

Supplement: Supplementary file 4 — Additional file 4. Funnel plot of the 28- or 30-day and 60-day mortality in comparison between corticosteroid treatment and control in early ARDS. [file 40560_2020_510_MOESM4_ESM.docx]

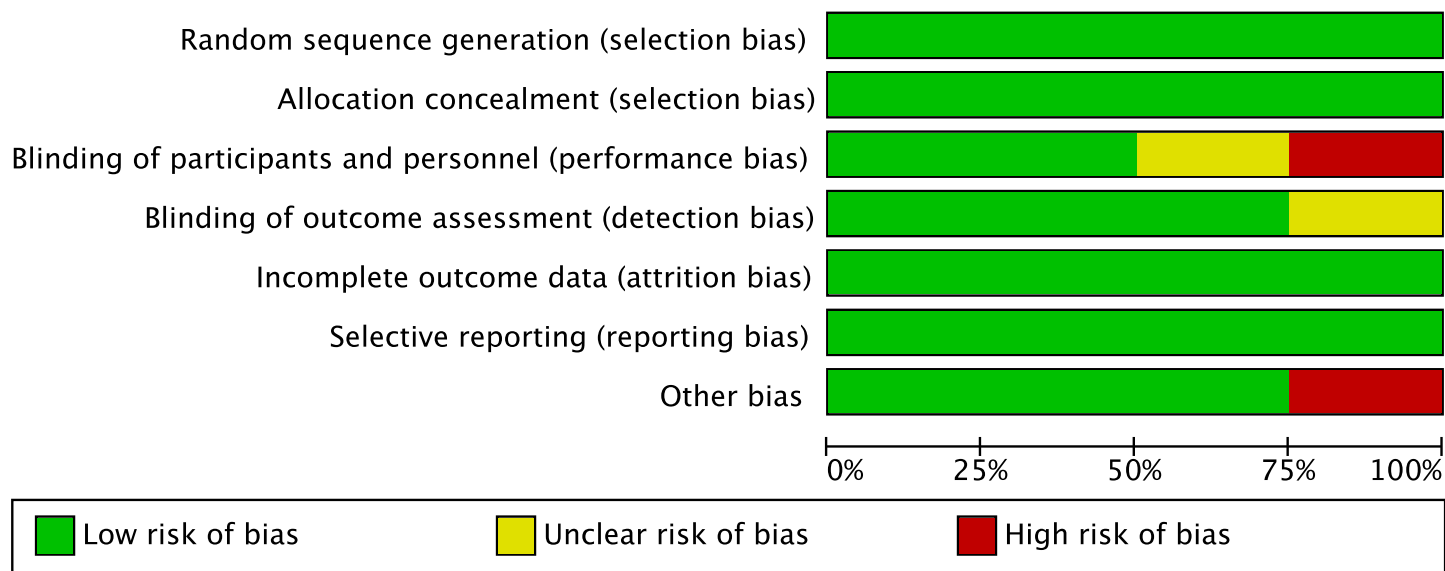

Supplement: Supplementary file 6 — Additional file 6. Risk of bias graph. [file 40560_2020_510_MOESM6_ESM.pdf]
