# Supplementary material for: Corticosteroid treatment for early acute respiratory distress syndrome: a systematic review and meta-analysis of randomized trials
Source: J Intensive Care. 2020 Dec 7;8:91. doi: 10.1186/s40560-020-00510-y (PMC7720037; doi:10.1186/s40560-020-00510-y)
Supplement: Supplementary file 5 — Additional file 5. Risk of bias summary. [file 40560_2020_510_MOESM5_ESM.pdf]

	Random sequence generation (selection bias)	Allocation concealment (selection bias)	Blinding of participants and personnel (performance bias)	Blinding of outcome assessment (detection bias)	Incomplete outcome data (attrition bias)	Selective reporting (reporting bias)	Other bias
Annane 2006	+	+	+	+	+	+	+
Meduri 2007	+	+	?	?	+	+	+
Tongyoo 2016	+	+	+	+	+	+	+
Villar 2020	+	+	−	+	+	+	−
